# Assessment of shared alleles in drought-associated candidate genes among southern California white oak species (*Quercus* sect. *Quercus*)

**DOI:** 10.1186/s12863-018-0677-9

**Published:** 2018-10-01

**Authors:** Signem Oney-Birol, Sorel Fitz-Gibbon, Jin-Ming Chen, Paul F. Gugger, Victoria L. Sork

**Affiliations:** 1 Department of Molecular Biology and Genetics, Faculty of Arts and Sciences, Burdur Mehmet Akif Ersoy University, 15030 Burdur, Turkey; 2 Institute of Genomics and Proteomics, University of California, Los Angeles, CA 90095 USA; 3 Key Laboratory of Aquatic Botany and Watershed Ecology, Wuhan Botanical Garden, Chinese Academy of Sciences, Wuhan, 430074 Hubei China; 4 Department of Ecology and Evolutionary Biology, University of California, Los Angeles, CA 90095-7239 USA; 5 Appalachian Laboratory, University of Maryland Center for Environmental Science, Frostburg, MD 21532 USA; 6 Institute of the Environment and Sustainability, University of California, Los Angeles, CA 90095-1496 USA

**Keywords:** Adaptation, Candidate genes, Drought stress, Hybridization, Introgression, RNAseq, Transcriptome, *Quercus* spp.

## Abstract

**Background:**

Hybridization and introgression are common phenomena among oak species. These processes can be beneficial by introducing favorable genetic variants across species (adaptive introgression). Given that drought is an important stress, impacting physiological and morphological variation and limiting distributions, our goal was to identify drought-related genes that might exhibit patterns of introgression influenced by natural selection. Using RNAseq, we sequenced whole transcriptomes of 24 individuals from three oaks in southern California: (*Quercus engelmannii*, *Quercus berberidifolia*, *Quercus cornelius-mulleri*) and identified genetic variants to estimate admixture rates of all variants and those in drought genes.

**Results:**

We found 398,042 variants across all loci and 4352 variants in 139 drought candidate genes. STRUCTURE analysis of all variants revealed the majority of our samples were assignable to a single species, but with several highly admixed individuals. When using drought-associated variants, the same individuals exhibited less admixture and their allele frequencies were more polarized between Engelmann and scrub oaks than when using the total gene set. These findings are consistent with the hypothesis that selection may act differently on functional genes, such as drought-associated genes, and point to candidate genes that are suggestive of divergent selection among species maintaining adaptive differences. For example, the drought genes that showed the strongest bias against engelmannii-fixed oak variants in scrub oaks were related to sugar transporter, coumarate-coA ligases, glutathione S-conjugation, and stress response.

**Conclusion:**

This pilot study illustrates that whole transcriptomes of individuals will provide useful data for identifying functional genes that contribute to adaptive divergence among hybridizing species.

**Electronic supplementary material:**

The online version of this article (10.1186/s12863-018-0677-9) contains supplementary material, which is available to authorized users.

## Background

The availability of genetic variants for evolutionary response of local populations to environmental change will depend on existing genetic variation, new mutations, gene flow among populations, or in some cases, interspecific gene flow. In many plant taxa, hybridization and backcrossing can lead to introgression of alleles from one species into the gene pool of another [1, 2]. As a result, it is possible for adaptive genetic variation to cross species boundaries [3–5] This interspecific gene flow can lead to adaptive evolution to novel ecological contexts [3–6]. In contrast, divergent selection can act to favor species-specific adaptive alleles and select against introgression at ecologically important loci [7–9], after initial F1 hybrid formation or across generations of backcrosses. Nonetheless, a first step in understanding whether introgression could enhance evolutionary response is to look for alleles shared across species.

Oaks (*Quercus* spp.) represent an excellent system to investigate selection favoring or acting against introgressed alleles [10] because of their well-known ability to hybridize but maintain ecological distinctiveness [11–14]. Many studies have documented the responses of *Quercus* species to drought [15–18]. Because so many *Quercus* species are adapted to drought-prone environments by ability either to avoid or tolerate water stress or both [19], introgression of genetic variants underlying these traits may provide the genetic material for species to adapt to climate change.

The overall goal of this paper is to examine a set of previously identified drought-associated genes that might be useful for future studies of climate-adaptive introgression and divergence. The study system is comprised of three sympatric species of white oaks (Section *Quercus*) found in southern California: one tree oak *Quercus engelmannii* and two drought-tolerant scrub oaks, *Quercus berberidifolia* and *Quercus cornelius-mulleri*. Engelmann oak is distantly related to the two recently diverged California endemic scrub white oak species [20–23]. These three species have overlapping distributions, but with different habitat preferences within southern California, a geographic region with a history of high temperatures and low rainfall [24]. We have evidence of contemporary hybridization among all three species [25] as well as ancient introgression between *Q. engelmannii* and *Q. berberidifolia* [26]. Using a sample of 24 study specimens representing the three species and putative hybrids sampled throughout the southern California region, we identified sequence variants across all expressed genes based on RNA-Seq data [27, 28] that were mapped to a previously published reference transcriptome for a related oak, *Quercus lobata* [29]. First, we described the overall admixture to assign species identity across the 24 individuals. Second, using variants derived from drought-associated genes that were identified through other studies [30–32], we examined the admixture to compare with the admixture based on the entire set of variants. Third, we looked for evidence of selection by testing whether alleles fixed in *Q. engelmannii* that are shared by a subset of the scrub oak species are less frequent in the two drought-tolerant scrub oaks when the variants are associated with drought genes compared to variants across all genes. Finally, we described the functions of drought associated genes that were outliers in the previous analysis.

## Methods

### Study specimen and sampling

The study species are three oak species of southern California that are sometimes sympatric with each other in many parts of their ranges [26, 33, 34]: *Q. engelmannii*, *Q. berberidifolia* and *Q. cornelius-mulleri*. Engelmann oak is native to southern California, USA and northern Baja California, Mexico), with the core of the species’ distribution range in San Diego County (USA), where the species is generally distributed in scattered patches often consisting of a few individuals [33, 35]. Engelmann oak is associated with more mesic local habitats than the southern California scrub oaks [24] and is more closely related to a Mexican/Arizona group of white oaks then to the scrub oaks [20]. The scrub oaks, *Q. berberidifolia* and *Q. cornelius-mulleri*, are both endemic to California and northern Baja California [20], but they belong to different genetic clusters within the white scrub oak complex [23, 26, 36]. *Q. berberidifolia* is widely distributed from northern California to Baja California and overlaps extensively but not completely the entire geographical range occupied by Engelmann oak [24, 33]. In contrast, *Q. cornelius-mulleri*, has a distribution restricted to southern California and northern Baja California usually in desert ecosystems, located more inland than *Q. berberidifolia* [24, 33]. They are known to hybridize with each other and hybrids show intermediate leaf traits and tend to be more shrub-like than tree-like [24, 33, 37].

**Fig. 1:**
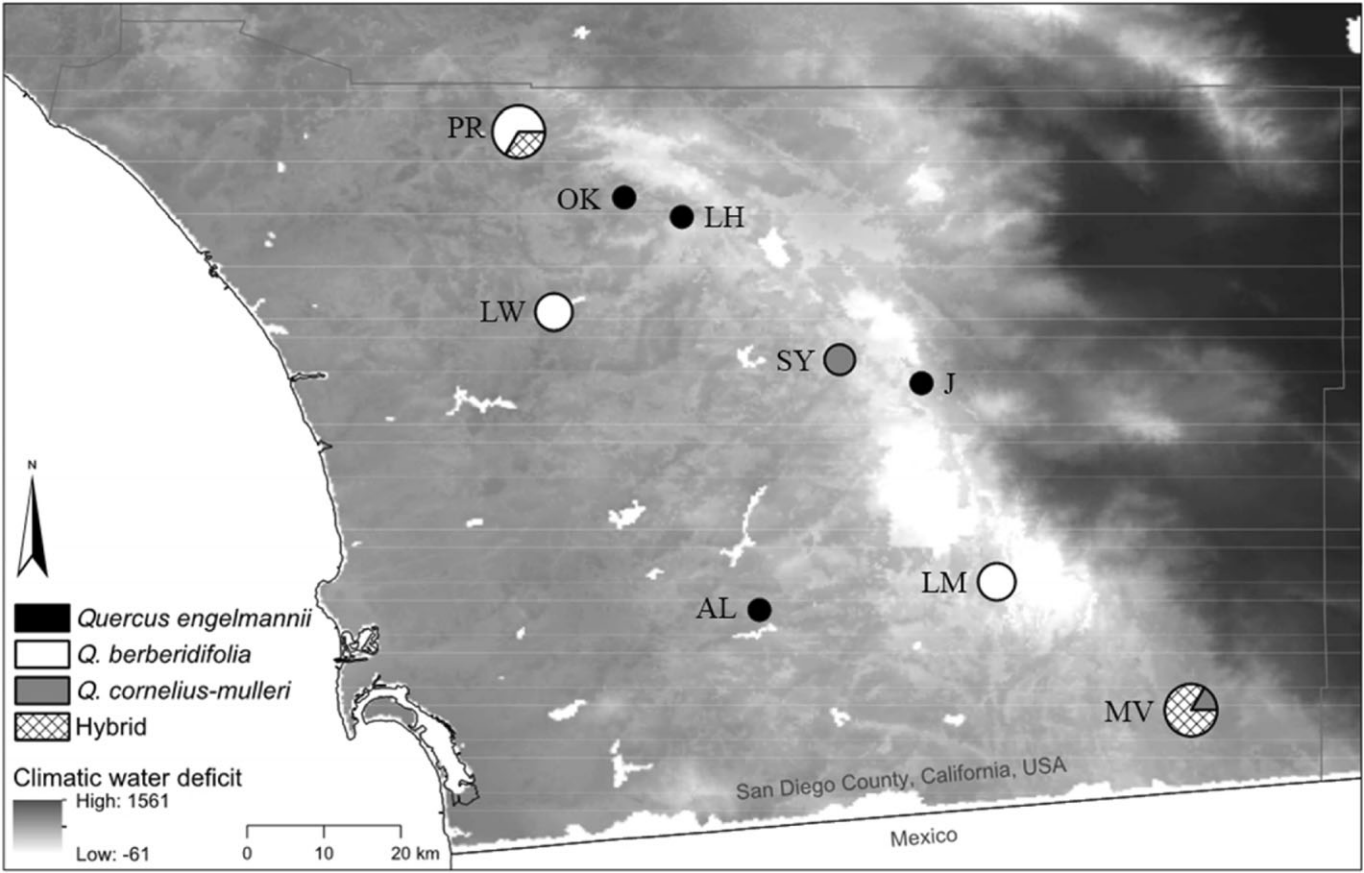
Map of region with location of sets of individuals from three species of oaks—*Q. berberidifolia*, *Q. cornelius-mulleri*, *Q. engelmannii*—and their hybrids found within southern California. Size of circle indicates number of individuals sampled. (See Table 1 for details of localities)

This study includes 24 individuals sampled from 9 different localities of Southern California (Table 1, Fig. 1). In some sites, only one species was present so it was intended that the individuals would represent putatively “pure” species, while in other sites more than one species was present creating the opportunity for contemporary hybridization. Nixon [20] includes a hybrid species between Engelmann and Muller oaks, Q. ×acutidens, but we did not attempt to determine whether the samples collected for this study could be considered as part of that nothospecies. Preliminary species and hybrid identifications of field samples were made by VLS and confirmed based on genetic information from the transcriptomes, which provided the bases of final assignments to species or hybrid. Voucher specimens are available through VLS.

**Table 1:** Localities of 24 individuals sampled from three southern California oak species: *Q. berberidifolia*, *Q. cornelius-mulleri*, and *Q. engelmannii*

Location	Sample IDs	Latitude	Longitude	Altitude (m)
Pala Reserve	8, 20, 21, 22, 23, 24	33° 22.53’	− 117° 02.71’	267
Oak Knoll	19	33° 17.92’	− 116° 55.29’	713
Lake Henshaw	18	33° 16.56’	−116° 51.24’	711
Lake Wohlford	7, 15, 16	33° 09.87’	−117° 00.23’	256
Santa Ysabel	1, 6	33° 06.55’	−116° 40.16’	999
Julian	14	33° 04.90’	−116° 34.42’	1683
Laguna Mountain	9, 13, 17	32° 50.97’	−116° 29.14’	1516
Alpine	2	32° 48.99’	−116° 45.80’	595
McCain Valley	3, 4, 5, 10, 11, 12	32° 41.95’	−116° 15.50’	1114

Localities are presented from northern to southern latitudes

### RNA extraction and sequencing

Fresh young leaf samples were frozen on dry ice in the field and stored at − 80 °C until total RNA extraction. Preliminary RNA precipitation [29, 30] as performed prior to total RNA extraction with the Qiagen RNeasy Plant Mini Kit protocol with DNase treatment (Qiagen, Hilden, Germany). RNA-Seq libraries with insert length 100–380 bp (mode = 170 bp) were prepared from 4 μg of RNA using an Illumina TruSeq RNA Sample Prep Kit. Each library was uniquely tagged using 12 TruSeq indexed adapters (numbers 1–12) to enable 12-plexing of samples in each Illumina HiSeq 2000 lane.

### Variant calling

We aligned a minimum of 13.8 million 50 bp reads per sample to our *Q*. *lobata* reference transcriptome [29] using BWA MEM [38]. Prior to mapping, the reference transcriptome was concatenated into 90 contigs of approximately 1 Mb in length. The original contigs were ordered by length and separated by runs of 200 Ns. This concatenation facilitates the analysis because GATK [39] and PLINK [40] were not designed for use with large numbers of reference contigs. GATK 3.2–2 [39] was used for variant discovery and filtering with the following tools, HaplotypeCaller, VariantFiltration and SelectVariants. To determine appropriate parameters for hard filtering with VariantFiltration, we inspected variant calls along with the read alignments using IGV [41]. The following cutoffs were applied: FS > 30.0, QD < 2.0 and QUAL < 30. Variants from the smallest contigs (737 bases and below) were discarded, leaving 858,564 variants on the first 58 of the 1 Mb concatenated contigs. The ratio of Ts/Tv for these variants was 1.76. The vcftools 0.1.15 package [42] was used to remove sites with mean depth coverage of less than 5 (−-min_meanDP 5) and with more than 5% of samples with missing data (−-max-missing 0.95) leaving 398,042 variants (Ts/Tv = 1.87). For STRUCTURE analysis, LD pruning was applied for *r*^2^ correlations above 0.1 within 50 bp windows, sliding by 5 bp using PLINK v1.90b3.36 [40] (indep-pairwise, based on correlations between genotype allele counts), leaving 30,809 variants (Ts/Tv = 1.93). For our allele frequency analysis, variants were limited to those for which a single allele was found across the four *Q. engelmannii* samples, leaving 219,407 variants (Ts/Tv = 1.85). The bedtools [43] package was used to facilitate allele frequency analyses.

### Drought gene selection

We selected 139 drought candidate genes for oaks that were identified in the literature [30, 32, 44] and located on the *Q. lobata* reference transcriptome [29].

### Structure analysis

**Fig. 2:**
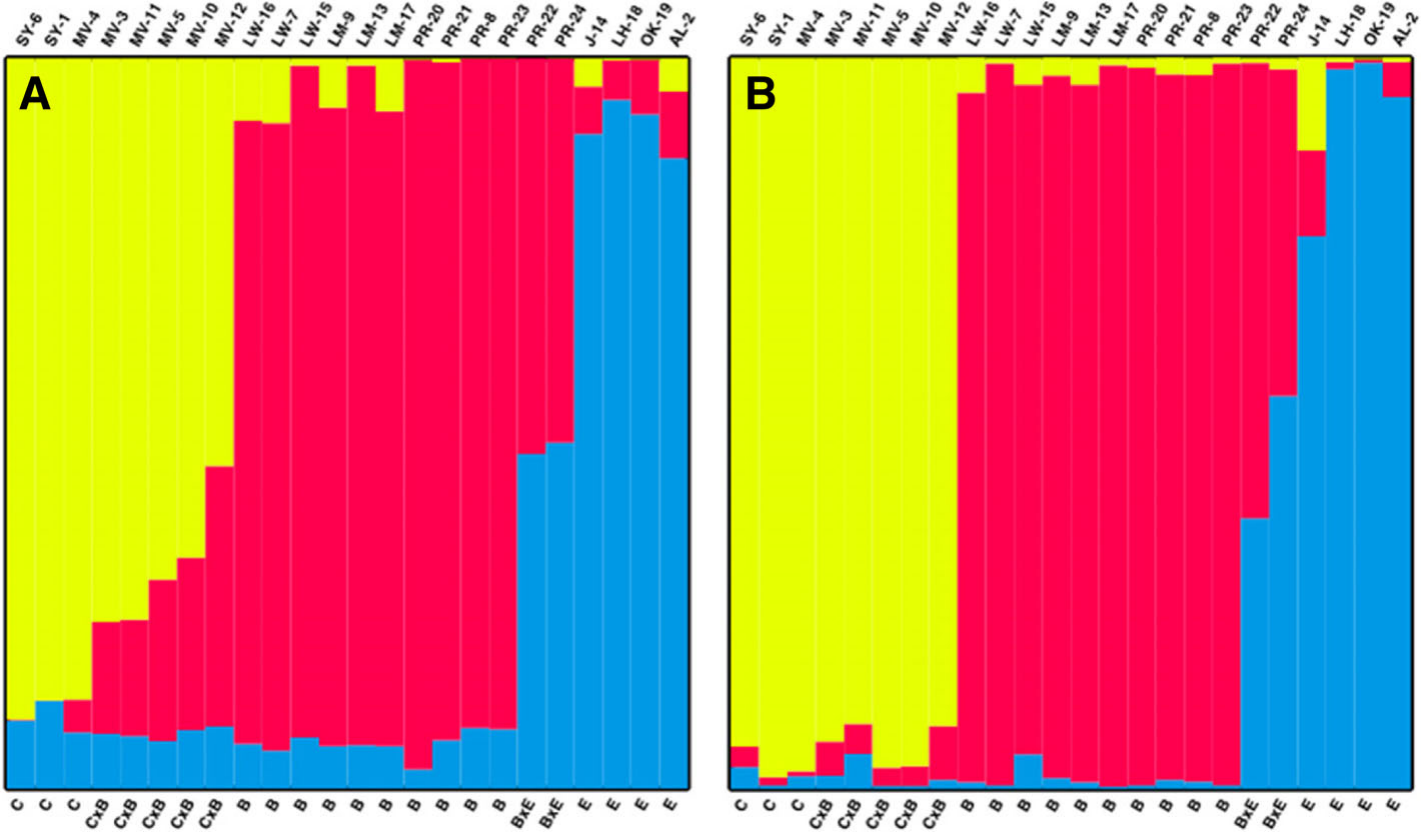
Assignment of species ancestry of 24 individuals collected from three species —*Q. berberidifolia*, *Q. cornelius-mulleri*, *Q. engelmannii*— across southern California, using STRUCTURE (K = 3). (**a**) Ancestry assignments are based on 219,407 LD-pruned variants across all sequences (**b**). Ancestry assignments are based on 30,809 LD-pruned variants associated with drought-related genes

Based on morphology of the samples that were assigned to the three species, we expected three genetic clusters. To test this hypothesis, we ran structure with *K* values from 1 to 9. The results were imported to Structure Harvester [45] to choose the “optimal” number of clusters following the methods of Evanno et al. [46]. STRUCTURE [47] was run on the filtered, LD pruned variants. We ran 10,000 burn-in and 20,000 experimental repetitions. Population label information was not used in the inference. The degree of admixture parameter, α, was inferred from the data. The allele frequency prior was estimated with preliminary runs (inferalpha = 0) on the full set of variants and set at 0.66 for all further runs, and correlated allele frequencies were assumed (freqscorr = 1). All other parameters were default. Membership coefficients for each sample were plotted using Distruct 1.1 [48] (Fig. 2).

**Fig. 3:**
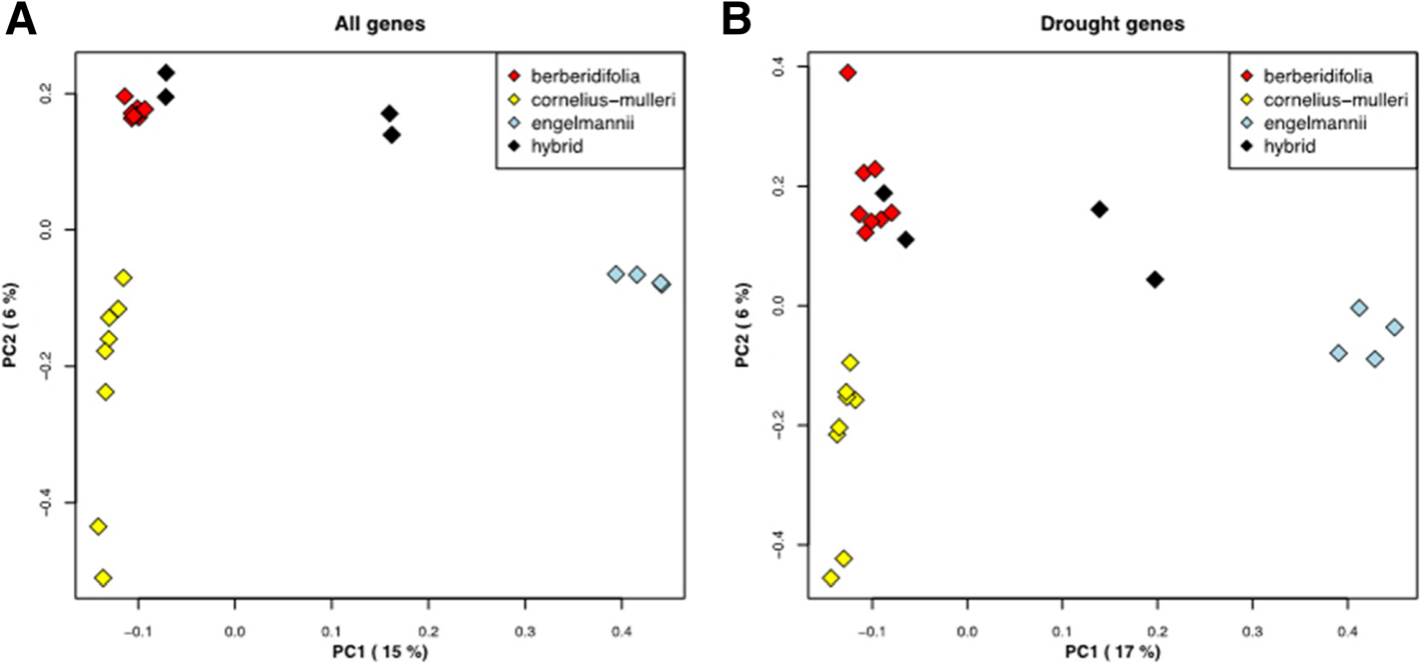
Principal component analysis of 24 individuals from three species using same data as Fig. 2. (**a**) Variants associated with all genes. (**b**) Variants associated with drought-related genes. Hybrids are identified based on STRUCTURE results

As a complement to STRUCTURE, we also examined the relationships among species using principal component analysis, which illustrates relationships among samples without assumptions about the assignment to species. For the PCA we used the R package SNPRelate [49], limiting the analysis to the 378,489 biallelic variants for all genes (Fig. 3a) and 4138 biallelic variants for the drought genes (Fig. 3b).

### Allele frequency analysis

We examined evidence of species-specific selection against drought gene introgression by selecting variants that are “fixed” in *Q. engelmannii* samples and variable in the scrub oak samples. We used quality-filtered, biallelic variant loci for which a single allele was found across the four *Q. engelmannii* samples. Four samples are not sufficient to determine fixation in the species, but for simplicity we refer to these loci as “*engelmannii*-fixed”. For each *engelmannii*-fixed variant locus, we measured the fraction of the *engelmannii*-fixed variant in the 20 scrub oak samples, i.e., the minimum of zero *Q. engelmannii* alleles = 0, and the maximum of 40 *Q. engelmannii* alleles = 1. We repeated this measure for the subset of *engelmannii*-fixed loci found in the 139 drought genes. We tested the significance of the increase in low *Q. engelmannii* allele frequencies by comparing the proportion of low *Q. engelmannii* loci in our drought genes with the same from 1000 randomly drawn sets of 139 genes. These random sets were drawn from the 13,159 transcriptome genes with the highest confidence annotations (i.e., those including 5′- and 3′-UTR, start and stop codons and no predicted introns).

### Functional descriptions of outlier genes

From the set of *engelmannii*-fixed variants, we selected those with less than 10% of the *Q. engelmannii* allele across the scrub oaks as outliers and identified overlapping drought genes to be functionally described. Additional information about putative functions were identified using BLASTX via the Gramene Server [50] to identify *Arabidopsis* homologs of the *Q. lobata* transcriptome contigs. Links for the top matching *Arabidopsis* gene were followed to the UniProtKB/Swiss-Prot server [51], from which protein names and protein function descriptions were taken. Most of the *Q. lobata* genes matched the *Arabidopsis* genes at greater than 80% amino acid identity and close to full length. All were greater than 58% identical (Table 2).

**Table 2:** Drought genes that contain variants that are “fixed” in *Q. engelmannii* and at low frequency and under-represented across 20 scrub oak samples

Gene (transcriptome contig)	Biallelic variants	*engelmannii*-fixed variants	Scrub oak variants with less than 10% e*ngelmannii*	% Identity to A*rabidopsis* protein	Uniprot Protein Name	Gene	Uniprot Function Description
m01oak10430CC	163	138	8	80%	CHY-type/CTCHY-type/RING-type Zinc finger protein	None	none
m01oak03512CC	71	55	7	71%	Monosaccharide-sensing protein 3	MSSP3	Sugar transport
m01oak02926cC	109	42	7	87%	Phosphoenolpyruvate carboxylase 1	PPC1	Through the carboxylation of phosphoenolpyruvate (PEP) it forms oxaloacetate, a four-carbon dicarboxylic acid source for the tricarboxylic acid cycle. Contributes probably to the adaptation to inorganic phosophate (Pi) deprivation
m01oak01922jc	71	49	5	92%	Cellulose synthase A catalytic subunit 2	CESA2	Catalytic subunit of cellulose synthase terminal complexes (‘rosettes’), required for beta-1,4-glucan microfibril crystallization, a major mechanism of the cell wall formation. Involved in the primary cell wall formation.
m01oak00521CC	61	53	3	87%	Heat shock 70 kDa protein 10, mitochondrial;Mitochondrial HSO70 2 isoform 2	HSP70	In cooperation with other chaperones, Hsp70s stabilize preexistent proteins against aggregation and mediate the folding of newly translated polypeptides in the cytosol as well as within organelles. These chaperones participate in all these processes through their ability to recognize nonnative conformations of other proteins. They bind extended peptide segments with a net hydrophobic character exposed by polypeptides during translation and membrane translocation, or following stress-induced damage (By similarity).
m01oak09381CC	22	21	2	81%	Amino acid permease 3	AAP3	Amino acid-proton symporter. Stereospecific transporter with a broad specificity for GABA, tryptophan and both neutral and basic amino acids. High affinity transport of cationic amino acids.
m01oak03200CC	66	46	2	79%	Polyol transporter 5	PLT5	Plasma membrane broad-spectrum sugar-proton symporter. Mediates the uptake of linear polyols such as sorbitol, xylitol, erythritol or glycerol. Can transport the cyclic polyol myo-inositol and different hexoses, pentoses (including ribose), tetroses and sugar alcohols.
m01oak01473cC	42	33	1	77%	Aspartic proteinase A1	APA1	Involved in the breakdown of propeptides of storage proteins in protein-storage vacuoles (By similarity). Possesses aspartic protease activity in vitro
m01oak03575jC	32	29	1	88%	Cryptochrome-1	CRY1	Photoreceptor that mediates primarily blue light inhibition of hypocotyl elongation and photoperiodic control of floral initiation, and regulates other light responses, including circadian rhythms, tropic growth, stomata opening, guard cell development, root development, bacterial and viral pathogen responses, abiotic stress responses, cell cycles, programmed cell death, apical dominance, fruit and ovule development, seed dormancy, and magnetoreception
m01oak00924cC	53	34	1	84%	Auxin-responsive protein IAA8	IAA8	Aux/IAA proteins are short-lived transcriptional factors that function as repressors of early auxin response genes at low auxin concentrations. Repression is thought to result from the interaction with auxin response factors (ARFs), proteins that bind to the auxin-responsive promoter element (AuxRE). Formation of heterodimers with ARF proteins may alter their ability to modulate early auxin response genes expression
m01oak01539cC	15	10	4	84%	Translation initiation factor SUI1 family protein (TAIR database)	AT5G11900	none

## Results and discussion

Using the reference transcriptome for a related oak species, *Q. lobata* [29], we aligned RNA-seq data for 24 individuals and identified 398,042 variants including 12,068 indels and 358,974 SNPs. After LD pruning, 1061 indels and 29,748 SNPs remained. We examined 139 drought-associated genes for introgression, which are listed in Additional file 1: Table S1. The transcriptome contigs for these genes had 4352 variants, with 330 remaining after LD pruning.

Based on the LD pruned data for all contigs/genes, we found support for three genetic clusters (Additional file 2: Figure S1; Table S2). None of our samples appear to have ancestry from only one species (Fig. 2a), although most trees could be assigned to one primary species that corresponded to our field identifications. We cannot explain why all samples included at least some genetic variation that was assigned to *Q. engelmannii* (Fig. 2a). It might be that shared genetic variation to all the species is assigned statistically to *Q. engelmannii* when it may be the signature of common ancestor or ancient introgression. The findings also indicate that two of the scrub oak samples (PR-22 and PR-24) are hybrids of *Q. engelmannii* and *Q. berberidifolia*, one sample (MV-12) is almost 50% from each scrub oak species and a fourth sample (MV-10) also shows marked admixture from both species. Several other *Q. cornelius-mulleri* samples included more than 10% of their genetic ancestry from *Q. berberidifolia* (MV-3, MV-5, MV-11). These patterns of assignments are likely indicative of introgression among the scrub oaks, but we cannot rule out common ancestry. Phylogenetic analysis indicates that these somewhat sympatric species are in different clades, but only recently diverged [26, 36].

The STRUCTURE analysis of assigned ancestry of the drought genes alone indicates similar patterns of introgression to those of the whole variant data set. One notable difference is that the five hybrids of *Q. cornelius-mulleri* and *Q. berberidifolia*, which show admixture of approximately 20--40% based on all variants (Fig. 2a), seem to be almost pure *Q. cornelius-mulleri* when looking at the drought genes alone (Fig. 2b). One must interpret these findings cautiously because the admixture analysis when based on drought genes has a reduced sample size of 330 variants compared to the 30,000+ variants across all sequences. Within the drought genes all the samples showed less admixture, which raises the question of whether selection is acting against variants not enhancing the fitness of a given species occurring within its own niche. The outcome is to make each species appear to have less introgression than indicated by the full set of variants. Because an alternative explanation for the ancestry patterns based on drought genes is sampling error, larger sample sizes would allow the permutation tests needed to test whether the pattern we observe is due to chance.

The PCA plots indicate differences in the relationships among individuals based on the total gene and drought gene sets (Fig. 3). The analyses for both samples of variants identifies the same two likely F_1_-hybrids between *Q. engelmannii* and *Q. berberidifolia*, but the scrub oak hybrids suggested by the STRUCTURE analysis cluster closely with *Q. berberidifolia* samples, suggesting that the introgression we observe is more likely to be due to admixture between *Q. engelmannii* and *Q. berberidifolia*, even though when looking at the distribution of variants on a one-by-one basis, it is not easy to say from which scrub oak they are shared. These findings are consistent with a concurrent study that demonstrated evidence of ancient introgression between the scrub white oaks and *Q. engelmannii* was largely due to shared alleles between *Q. engelmannii* and *Q. berberidifolia* and not with *Q. cornelius-mulleri* [26].

**Fig. 4:**
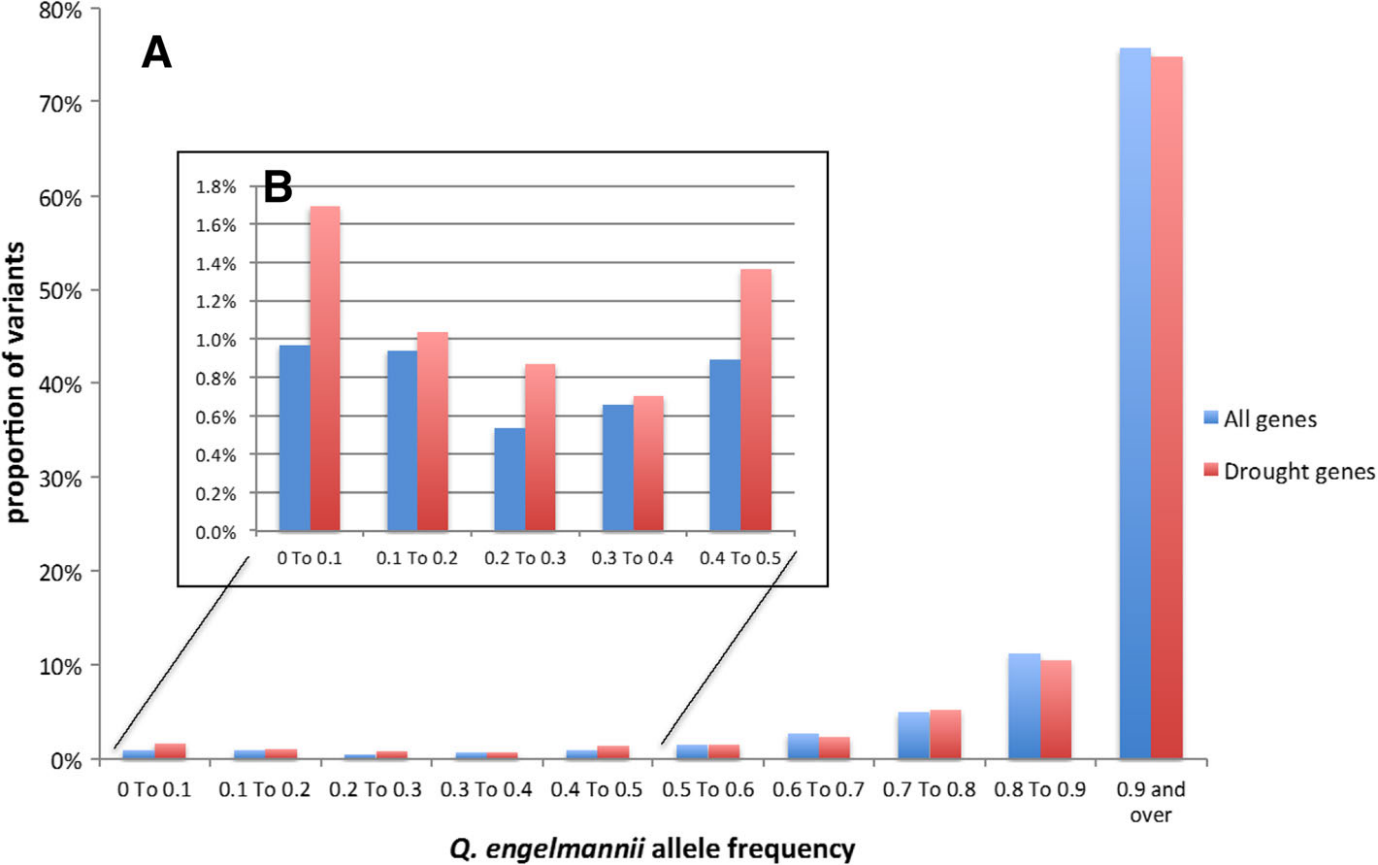
(**a**) Frequencies of *Q. engelmannii*-fixed alleles in the 20 scrub oaks: The frequencies of *engelmannii*-fixed alleles for each variant site is calculated as the number of *Q. engelmannii*-fixed alleles in the scrub oaks divided by the total number of alleles across all scrub oaks at that locus, usually 40 depending on missing data. The proportion of variants falling into each allele frequency bin is reported as a percentage of the total number of variants, 219,407 for all genes and 2421 for the drought genes. These variant sets are non-LD-pruned *engelmannii*-fixed biallelic loci with no more than 5% missing data. (**b**) Allele frequencies less than 0.5. Overall 8746 (4.0%) sites have a *Q. engelmannii*-fixed allele frequency in scrub oaks of 0.5 or less, and 137 (5.7%) drought gene variant sites have a *Q. engelmannii*-fixed allele frequency in scrub oaks of 0.5 or less. The difference between all tested bins of low allele frequencies mostly reversed at high frequency bins

To find evidence that selection is acting on drought-associated genetic variants, we looked for a decrease of allele number from the more mesic *Q. engelmannii* found within genes associated with drought compared to all genes. To do this, we identified variants that are “fixed” in *Q. engelmannii*, i.e., having the same allele for all eight *Q. engelmannii* haplotypes (across four samples), and looked at the frequencies of these alleles across the scrub oaks. We discovered 219,407 *engelmannii*-fixed loci of which 2422 were located within the 139 drought genes. As one would expect since most variants have low minor allele frequencies, the vast majority of the “*engelmannii*-fixed” alleles were shared among greater than 90% of the scrub oaks. However, “*engelmannii*-fixed” alleles that are low frequency (defined as less than 50%) in the scrub oaks (2121 SNPs in 726 genes) are underrepresented in the scrub oaks compared to low-frequency alleles in the total gene data set (Fig. 4). This result is apparent in the left skew on the histogram of drought gene allele frequencies relative to other gene allele frequencies. The difference is seen across all tested bins of low allele frequencies (Fig. 4b) and is mostly reversed when looking at the high frequency bins. The observation that *Q. engelmannii* drought gene alleles are rarer in scrub oak than expected based on allele frequencies from other genes might suggest selection against *Q. engelmannii* alleles in the scrub oaks and thus adaptive divergence among species.

**Fig. 5:**
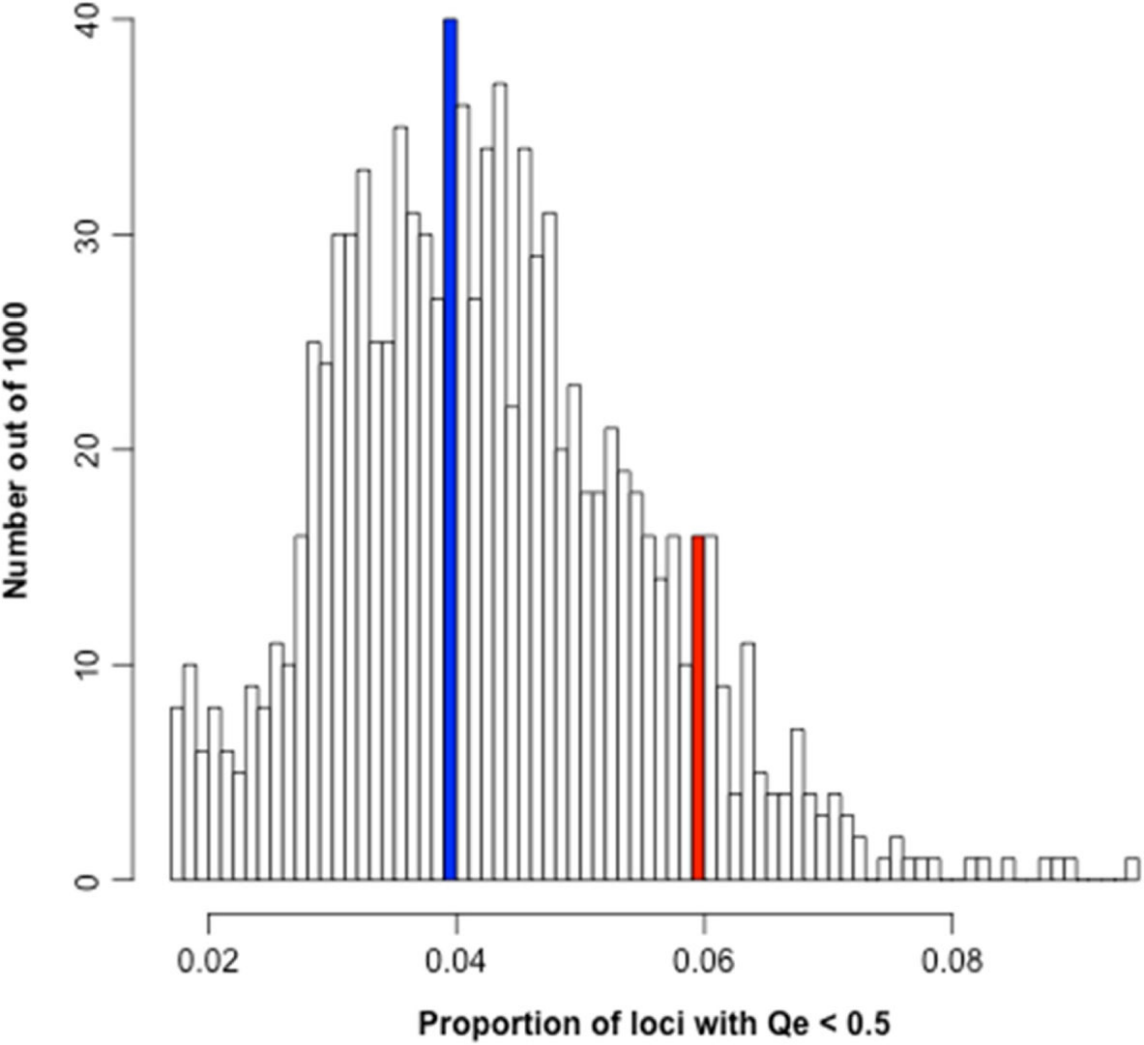
The proportion of variant sites with *Q. engelmannii* allele frequencies below 50% in the scrub oaks is shown in blue for all genes, red for the 139 drought genes and white for 1000 random sets of 139 genes

In order to test the significance of this difference, we compared the results to 1000 random sets of 139 genes (equivalent to the number of drought genes) (Fig. 5). We then examined the results with the proportion of low-frequency *engelmannii*-fixed alleles for all genes (shown in blue; Fig. 5) with the proportion for drought genes (shown in red) and the proportion for the 1000 sets of random genes (shown in white). We see a fairly normal distribution with the drought genes skewed to having a higher proportion of low frequency *Q. engelmannii* alleles. Only 105 out of 1000 random gene sets have a higher proportion of low frequency *Q. engelmannii* alleles, yielding *p* = 0.1. Thus, the analysis suggests that the scrub oaks are not significantly less likely to carry *Q. engelmannii* drought gene alleles than expected from patterns of overall allele sharing across the three species. The initial findings reported here are consistent with the notion that *Q. engelmannii* variants within the drought genes are rarer in the scrub oaks than expected and might suggest divergent selection among species at these ecological relevant loci.

Finally, we use these findings to narrow down our set of candidate genes potentially involved in drought resistance to a set of genes that vary across species and warrant further study. Drought-associated genes that are fixed across oaks and or that vary but with no species-specific signature will not allow analysis of adaptive introgression. However, drought genes that are species-species can be informative. Looking only at variants with less than 10% of the *Q. engelmannii* alleles found across the scrub oaks, we find 41 variants fall within 11 of the 139 candidate drought genes (Table 2). Two of our hybrid samples from one of the localities offered the opportunity to look for additional loci where the *engelmannii*-fixed alleles might show evidence that selection has acted against them: PR-22 and PR-24. For each of the two hybrid samples, we identified *engelmannii*-fixed loci where the hybrid is homozygous for the alternate allele, i.e., it has zero of the *Q. engelmannii* allele. Out of 219,407 *engelmannii*-fixed loci in our dataset, PR-22 had 1993 loci with zero of the *Q. engelmannii* allele, of which 33 were within 4 drought genes (Table 3), 2 of which were also identified in Table 2. PR-24 had 1863 loci with zero of the *Q. engelmannii* allele, of which 14 were within 5 drought genes (Table 4).

**Table 3:** Drought genes in hybrid PR-22 lacking variants found in *Q. engelmannii* allele, as candidates for genes involved in local adaptation to warm or dry habitats

Gene (transcriptome contig)	Biallelic variants	*engelmannii-*fixed variants	Variants with no *Q. engelmannii* allele in hybrid PR-22	% Identity to Arabidopsis	Uniprot Protein Name	Gene	Uniprot Function Description
m01oak02926cC (also in Table 2)	109	42	18	87%	Phosphoenolpyruvate carboxylase 1	PPC1	Through the carboxylation of phosphoenolpyruvate (PEP) it forms oxaloacetate, a four-carbon dicarboxylic acid source for the tricarboxylic acid cycle. Contributes probably to the adaptation to inorganic phosophate (Pi) deprivation
m01oak03200CC (also in Table 2)	66	46	11	79%	Polyol transporter 5	PLT5	Plasma membrane broad-spectrum sugar-proton symporter. Mediates the uptake of linear polyols such as sorbitol, xylitol, erythritol or glycerol. Can transport the cyclic polyol myo-inositol and different hexoses, pentoses (including ribose), tetroses and sugar alcohols.
m01oak01188Ct	7	5	1	58%	Yellow leaf specific gene 9	YLS9	Up-regulated in leaves during natural senescence
m01oak04613CC	43	12	3	68%	ACT domain-containing protein	ACR8	May bind amino acids; induced by abscisic acid (ABA), and cold and salt stresses

**Table 4:** Drought genes in hybrid PR-24 lacking variants found in *Q. engelmannii*, as candidates for genes involved in local adaptation to warm or dry habitats

Gene (transcriptome contig)	Biallelic variants	*engelmannii*-fixed variants	Variants with no *engelmannii* allele in hybrid PR-24	% Identity to Arabidopsis protein	Uniprot Protein Description	Gene	Uniprot Function Description
m01oak09012cC	61	47	6	80%	4-coumarate--CoA ligase-like 7	4CLL7	Contributes to jasmonic acid biosynthesis by initiating the beta-oxidative chain shortening of its precursors.
m01oak03358CC	56	24	5	81%	4-coumarate--CoA ligase 1	4CL1	Produces CoA thioesters of a variety of hydroxy- and methoxy-substituted cinnamic acids, which are used to synthesize several phenylpropanoid-derived compounds, including anthocyanins, flavonoids, isoflavonoids, coumarins, lignin, suberin and wall-bound phenolics
m01oak00399CT	12	9	1	63%	Glutathione S-transferase F7	GSTF7	May be involved in the conjugation of reduced glutathione to a wide number of exogenous and endogenous hydrophobic electrophiles and have a detoxification role against certain herbicides (also possible response to salt stress)

There were many interesting potential functions amongst this narrowed down set, which include several sugar transporters, two separate homologs to coumarate-coA ligases, two genes involved in glutathione S-conjugation, and several stress response genes. Other studies have found other genes such as FBA1 [52], BURP [53, 54], USP [55], GH16_XET [56], CYP707 gene family [57], HSF [58], AAI_LTSS [59] (Additional file 1: Table S1) to be involved in drought resistance that we would have expected to be important in this study but did not show species-specific signatures in this study. The genes we report these here so that future studies of local adaptation might use them for comparison.

## Conclusion

This exploratory study found evidence of modest admixture among two scrub white oak species and one tree oak based on a transcriptome-wide set of loci. When we examined a subset of drought-associated genes, variants that were most associated with the more mesic Engelmann oak were under-represented across the scrub oaks, which is consistent with the hypothesis that these variants are selected against by the local environment. Specifically, we identified eleven genes that may be examples of species-specific adaptation associated with drought. Of course, one limitation of this study is that some genes may be so extensively introgressed that we would not be able to distinguish them from shared common ancestry. Future work would be able to do so through larger samples sizes that can more sensitively identify more species-specific variants, and complementary analyses using genome-wide DNA sequence data that can test alternative demographic models that test for introgression while accounting for incomplete lineage sorting. Nonetheless, this study illustrates that the transcriptomes of individuals across hybridizing species have the potential to provide useful data to study adaptive introgression because they can identify functional genes associated with specific environmental factors.

## Additional files

Additional file 1: Candidate gene list associated with drought stress. **Table S1.** Candidate loci were taken from evoltree database depending on search tool for drought and hypoxia relates stress (target trait) and matched the sequences to the *Quercus lobata* reference transcriptome [29]. The information of gene names, protein locus and functions was taken from NCBI database based on given information in evoltree database [32]. (XLS 48 kb)

Additional file 2: Calculations of Structure Harvester and Evanno method results. **Figure S1.** Structure Harvester Results. Structure was run with settings as described in the methods section for all K values between 1 and 9. The results were imported to the online version of Structure Harvester [45], yielding support for K = 3 being the best approximation. **Table S2.** Based on Evanno et al. [46] method results. This table lists Delta K values for K values from 1 to 9 associated with samples that were assigned to the three species of oaks based on morphology. (DOCX 93 kb)

## Abbreviations

AL: Alpine
BWA: Burrows-Wheeler Aligner
FS: FisherStrand
J: Julian
LD: Linkage disequilibrium
LH: Lake Henshaw
LM: Laguna Mountain
LW: Lake Wohlford
MV: McCain Valley
OK: Oak Knoll
PCA: Principal component analysis
PR: Pala Reserve
QD: QualByDepth
SNPs: Single nucleotide polymorphisms
SY: Santa Ysabel
Ts/Tv: Transitions to tranversions
