# Supplementary material for: Assessment of shared alleles in drought-associated candidate genes among southern California white oak species (Quercus sect. Quercus)
Source: BMC Genet. 2018 Oct 1;19:88. doi: 10.1186/s12863-018-0677-9 (PMC6167808; doi:10.1186/s12863-018-0677-9)

**Assessment of shared alleles in drought-associated candidate genes among southern California white oak species (*Quercus* sect. *Quercus*)**

Signem Oney-Birol^1^*, Sorel Fitz-Gibbon^2^*, Jin-Ming Chen^3^, Paul F. Gugger ^4, 5^,

Victoria L. Sork^4,6^ *

^1^ Department of Molecular Biology and Genetics, Faculty of Arts and Sciences, Mehmet Akif Ersoy University, Burdur, 15030, Turkey

^2^ Institute of Genomics and Proteomics, University of California, Los Angeles, CA

^3^ Key Laboratory of Aquatic Botany and Watershed Ecology, Wuhan Botanical Garden, Chinese Academy of Sciences, Wuhan, Hubei 430074, China

^4^ Department of Ecology and Evolutionary Biology, University of California, Los Angeles, CA 90095-7239, USA

^5^ University of Maryland Center for Environmental Science, Appalachian Laboratory, Frostburg, MD 21532, USA

^6^ Institute of the Environment and Sustainability, University of California, Los Angeles, CA 90095-1496, USA

* Contributed equally to the manuscript

Corresponding authors: [vlsork@ucla.edu](mailto:vlsork@ucla.edu) , [sobirol@mehmetakif.edu.tr](mailto:sobirol@mehmetakif.edu.tr)

**Supplemental Information (Figure S1, Table S2)**

**Figure S1**. **Structure Harvester Results**. Structure was run with settings as described in the methods section for all K values between 1 and 9. The results were imported to the online version of Structure Harvester (Earl and vonHoldt 2011), yielding support for K=3 being the best approximation. (See Table S2).

**Table S2.** Based on Evanno et al. (2005), this table lists Delta K values for K values from 1 to 9 associated with samples that were assigned to the three species of oaks based on morphology. The highest Delta K indicates that the optimal number of genetic clusters is K=3, using Structure Harvester (Earl and vonHoldt 2011).


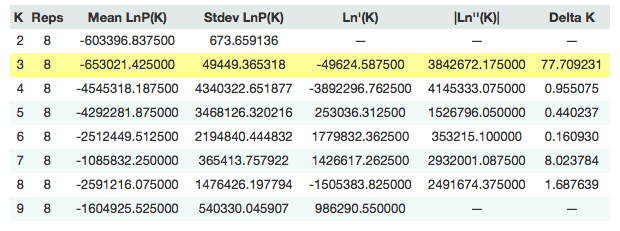

Supplement: Supplementary file 2 — Calculations of Structure Harvester and Evanno method results. Figure S1. Structure Harvester Results. Structure was run with settings as described in the methods section for all K values between 1 and 9. The results were imported to the online version of Structure Harvester [45], yielding support for K = 3 being the best approximation. Table S2. Based on Evanno et al. [46] method results. This table lists Delta K values for K values from 1 to 9 associated with samples that were assigned to the three species of oaks based on morphology. (DOCX 93 kb) [file 12863_2018_677_MOESM2_ESM.docx]
